# Transcriptomic profiling identifies a nucleotide metabolism-related signature with prognostic power in gliomas

**DOI:** 10.1016/j.tranon.2024.102068

**Published:** 2024-08-08

**Authors:** Ai-Qin Chen, Qi-Xuan Jiang, Yong-Jian Zhu, Qiang-Wei Wang

**Affiliations:** a Department of Neurosurgery, The Second Affiliated Hospital, Zhejiang University School of Medicine, Hangzhou 310009, China; b Key Laboratory of Precise Treatment and Clinical Translational Research of Neurological Diseases, Hangzhou 310009, China; c Clinical Research Center for Neurological Diseases of Zhejiang Province, Hangzhou 310009, China

**Keywords:** Nucleotide metabolism, Glioma, Prognosis, Signature, Tumor immunity

## Abstract

**Objective:**

Nucleotide metabolic reprogramming as a hallmark of cancer is closely related to the occurrence and progression of cancer. We aimed to comprehensively analyze the nucleotide metabolism-related gene set and clinical significance in gliomas.

**Methods:**

The RNA sequencing data of 702 gliomas from the Cancer Genome Atlas (TCGA) dataset were included as the training set, and the RNA sequencing data from the other three datasets (CGGA, GSE16011, and Rembrandt) were used as independent validation sets. Survival curve, Cox regression analysis, time-dependent ROC curve and nomogram model were performed to evaluate prognostic power of signature. R language was the main tool for bioinformatic analysis and graphical work.

**Results:**

Based on the expression profiles of nucleotide metabolism-related genes, consensus clustering identified two robust clusters with different prognosis. We then developed a nucleotide metabolism-related signature that was closely related to clinical, pathological, and genomic characteristics of gliomas. And ROC curve showed that our signature was a potential biomarker for mesenchymal subtype. Survival curve and Cox regression analysis revealed signature as an independent prognostic factor for gliomas. In addition, we constructed a nomogram model to predict individual survival. Finally, functional analysis showed that nucleotide metabolism not only affected cell division and cell cycle, but also was associated with immune response in gliomas.

**Conclusion:**

We developed a nucleotide metabolism-related signature to predict prognosis and provided new insights into the role of nucleotide metabolism in gliomas.

## Highlights

• Nucleotide metabolism-related gene set could classify the glioma patients with different prognosis.
• We developed a nucleotide metabolism-related signature to divide patients into high-risk or low-risk group.
• There were significant differences in clinical and pathological features, genomic alterations and prognosis between the two groups.
• Our signature was an independent prognostic indicator of gliomas, and we constructed a nomogram model for individualized survival prediction.
• Nucleotide metabolism was not only related to cell division and cell cycle, but also affected the immune microenvironment in gliomas.

## Introduction

Gliomas are the most common malignant primary brain tumor in adults and arise from glial or precursor cells [1]. In adults, the disease mainly consists of diffuse tumors ranging from grade 2 to 4. Currently, the treatment of gliomas requires a multidisciplinary approach, including surgical resection, radiotherapy, systemic treatment and supportive care [2]. Unfortunately, despite aggressive treatment, most patients with glioma have an extremely poor prognosis. In the most common type, glioblastoma (GBM, grade 4), only 5.5 % of patients survive more than five years [3]. In order to address the limitations of current treatment strategies, we need to further explore the pathogenesis of gliomas and develop new therapies to improve the prognosis of patients.

Nucleotides are necessary in various biological processes, both for DNA replication and RNA synthesis. Meanwhile, many metabolic central molecules are also nucleotides or contain nucleotide fragments, such as ATP, NADH, CoA, and so on [4]. In cancer, mutations and genomic aberrations that promote biosynthesis, together with changes in metabolic enzyme expression, lead to elevated levels of nucleotide synthesis [5]. Increased nucleotide metabolism is a hallmark of cancer and nucleotides are mainly used as raw materials for nucleic acid synthesis to support the uncontrolled proliferation of tumor cells [6]. In addition to their proliferative role in tumors, purine molecules have been well recognized as purinergic signaling ligands that mediate proliferation, differentiation and apoptosis of tumor cells [7]. And pyrimidine metabolism can also maintain epithelial-to-mesenchymal transition phenotype in tumors and influence metastasis [8]. Inhibition of nucleotide metabolic pathways has long been considered as a therapeutic strategy for cancer. Purine and pyrimidine analogs are used in cancer treatment, and they are incorporated into DNA and inhibit the activity of DNA polymerase [9]. Some classical drugs, such as 6-MP, methotrexate and 5-fluorouracil, are used for cancer treatment by inhibiting enzymes involved in nucleotide metabolism [10,11]. Exploring and clarifying the complex mechanism of nucleotide metabolism in tumors will facilitate the development of nucleotide metabolism-specific drugs and improve the survival and prognosis of tumors, including gliomas.

In this study, we comprehensively analyzed the nucleotide metabolism-related gene set and found that they could classify the glioma patients with different prognosis. Then we developed a nucleotide metabolism-related signature to divide patients into high-risk or low-risk group. There were significant differences in clinical and pathological features, genomic alterations and prognosis between the two groups. Our signature was an independent prognostic indicator of gliomas, and we constructed a nomogram model for individualized survival prediction. Functional analysis showed that nucleotide metabolism was not only related to cell division and cell cycle, but also affected the immune microenvironment in gliomas.

## Methods

### Patients and datasets

In the training set, the Cancer Genome Atlas (TCGA) dataset [[12], [13], [14], [15]] contained RNA sequencing data, somatic mutations, copy number alterations (CNAs), and clinical and pathological information from 702 glioma samples. 702 samples (173 GBM + 529 LGG) from TCGA dataset were downloaded from the UCSC Xena Project database (http://xena.ucsc.edu/, accessed on 19 Jul 2019). For mRNA data, RNA expression values were all log2 transformed, and filtered to remove low-variability genes (bottom 25 % removed, based on interdecile range). The validation sets contained our Chinese Glioma Genome Atlas (CGGA) dataset, GSE16011 dataset, and Rembrandt dataset. In our CGGA dataset, we have collected RNA sequencing data from 325 glioma samples, generated by the Illumina HiSeq platform. We have also collected clinical and pathological information from CGGA patients. Our CGGA dataset was approved by the Beijing Tiantan Hospital Capital Medical University Institutional Review Board (IRB KY2013-017-01) [16,17]. Two other validation datasets included 268 glioma samples from the GSE16011 microarray dataset (http://www.ncbi.nlm.nih.gov/geo/query/acc.cgi?acc=GSE16011) and 454 glioma samples from the Rembrandt microarray dataset (https://www.ncbi.nlm.nih.gov/geo/query/acc.cgi?acc=GSE108474).

### Consensus clustering

Two nucleotide metabolism-related gene sets (REACTOME_METABOLISM_OF_NUCLEOTIDES and MODULE_337) were obtained from the Molecular Signatures Database v7.4 (MSigDB, https://www.gsea-msigdb.org/gsea/msigdb/). After the elimination of duplicate genes, 155 nucleotide metabolism-related genes were used for the subsequent analysis. We identified the most variable genes by the median absolute deviation (MAD > 0.5) and consensus clustering analysis was performed with R package “ConsensusClusterPlus”.

### Gene signature identification

In the TCGA training set, univariate Cox regression analysis was performed to evaluate the prognostic value of nucleotide metabolism-related genes, and 115 genes related to survival (*p* < 0.05 by Wald test) were selected. With the selected genes, the Least Absolute Shrinkage and Selection Operator (LASSO) algorithm [18] generated a Cox model including 17 genes, which had the minimum average cross-validation error. Our signature risk score was developed from a linear combination of expression levels (expr) of 17 genes, which were weighted by their LASSO regression coefficients: Risk Score = (expr_gene1_ x coefficient_gene1_) + (expr_gene2_ x coefficient_gene2_) + … + (expr_gene17_ x coefficient_gene17_). The same formula and regression coefficients were used to calculate the risk score of three validation sets.

### Bioinformatic analysis

Univariate and multivariate Cox regression analysis were performed to evaluate the prognostic value of variables including our signature. Variance inflation factor (VIF) was used for collinearity diagnosis, and variables without collinearity (VIF < 5) were included in the regression analysis. Time-dependent ROC curve (timeROC) was applied to predict one-, three- and five-year overall survival with R package “timeROC” [19,20]. The individualized prediction model nomogram integrated signature and clinical indicators with R package “rms” [21]. Gene Ontology (GO) and Kyoto Encyclopedia of Genes and Genomes (KEGG) pathway enrichment analysis were performed with genes that were significantly positively correlated with signature (Pearson *R* > 0.6, *p* < 0.05) in DAVID Bioinformatics Resources 6.8 (https://david.ncifcrf.gov/). Gene set enrichment analysis (GSEA) was performed with R package “fgsea”. ESTIMATE algorithm was performed to calculate immune and stromal scores for gliomas [22]. CIBERSORT algorithm was performed to evaluate the proportion of 22 immune cell types in gliomas [23,24].

### Single-cell RNA sequencing

We collected two samples of primary IDH-wildtype glioblastoma patients from Beijing Tiantan Hospital, Capital Medical University. Fresh glioma samples collected during surgery were immediately transported to the laboratory and processed with enzyme digestion and red blood cell lysis to produce single-cell suspensions. According to the manufacture's introduction, the cDNA libraries were constructed with Chromium Single Cell 3′ Library and Gel Bead kit v2 (120267, 10x Genomics). The libraries were sequenced on Illumina HiSeq platform and the raw data was preprocessed with Cell Ranger pipeline (v3.0.2, 10X Genomics), mapped to the hg19 reference genome. Cells in gene-cell matrices with fewer than 200 transcripts and genes with fewer than two counts in two cells were filtered and removed. The matrix was then normalized such that the number of Unique Molecular identifiers (UMIs) in each cell was equal to the median UMI count across the dataset and log-transformed. Gene-barcode matrices were analyzed with the R package “Seurat” (version 5) [25]. Based on QC metrics in the standard pre-processing workflow for scRNA-seq data, we filtered cells that had unique feature counts over 4000 or less than 200, UMI counts over 30,000 and 20 % mitochondrial counts. To reduce the gene expression matrix to its most important features, we used principal component analysis (PCA) to decrease the dimensionality of the dataset. To visualize data in 2-D space, we passed the PCA-reduced data into UMAP (uniform manifold approximation and projection), a non-linear dimensional reduction method.

### Statistical analysis

R language (https://www.r-project.org/) was the main statistical and graphic environment in our study. Differences in variables between groups were assessed by Student's *t*-test or Chi-square test. The Kaplan-Meier survival curve was evaluated by log-rank test. Other R packages for drawing included pheatmap, gglpot2, pROC, ComplexHeatmap, and circlize. *p* value less than 0.05 was statistically significant.

## Results

### Identification of a nucleotide metabolism-related signature in gliomas

**Fig. 1:**
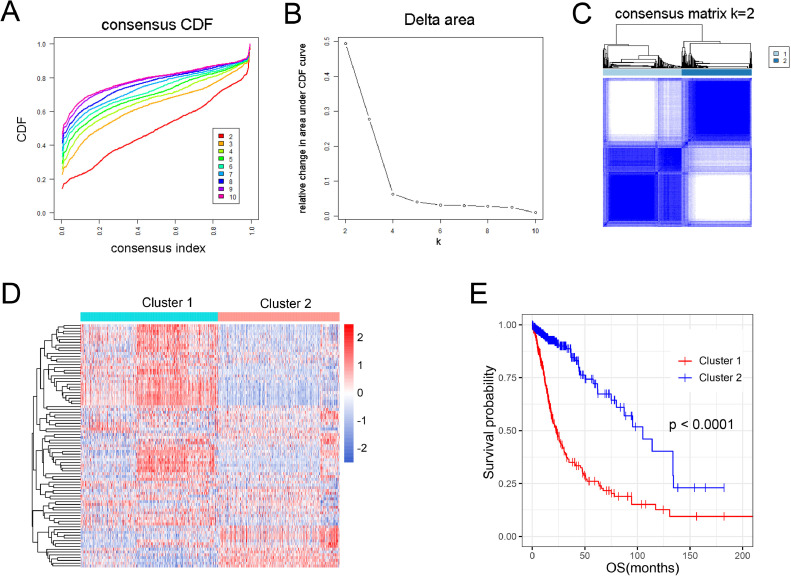
Consensus clustering of gliomas with nucleotide metabolism-related genes. (A) Consensus clustering cumulative distribution function (CDF) for *k* = 2 to *k* = 10. (B) Relative change in area under CDF curve for *k* = 2 to *k* = 10. (C) Consensus clustering matrix of 702 glioma samples from TCGA dataset for *k* = 2. (D) Heatmap of the two clusters defined by the nucleotide metabolism-related genes (MAD > 0.5). (E) Survival curve analysis of glioma patients in two clusters.

We first explored the relationship between nucleotide metabolism status and glioma prognosis. Consensus clustering was performed using nucleotide metabolism-related gene sets, and TCGA patients could be divided into two robust clusters (Fig. 1A-C). The heatmap showed that the expression of nucleotide metabolism-related genes was significantly different between the two clusters (Fig. 1D). Meanwhile, the survival curve showed that the overall survival of patients in cluster 1 was significantly shorter than that in cluster 2 (*p* < 0.05, Fig. 1E). These results indicated that nucleotide metabolism status was closely related to the prognosis of patients with glioma.

**Fig. 2:**
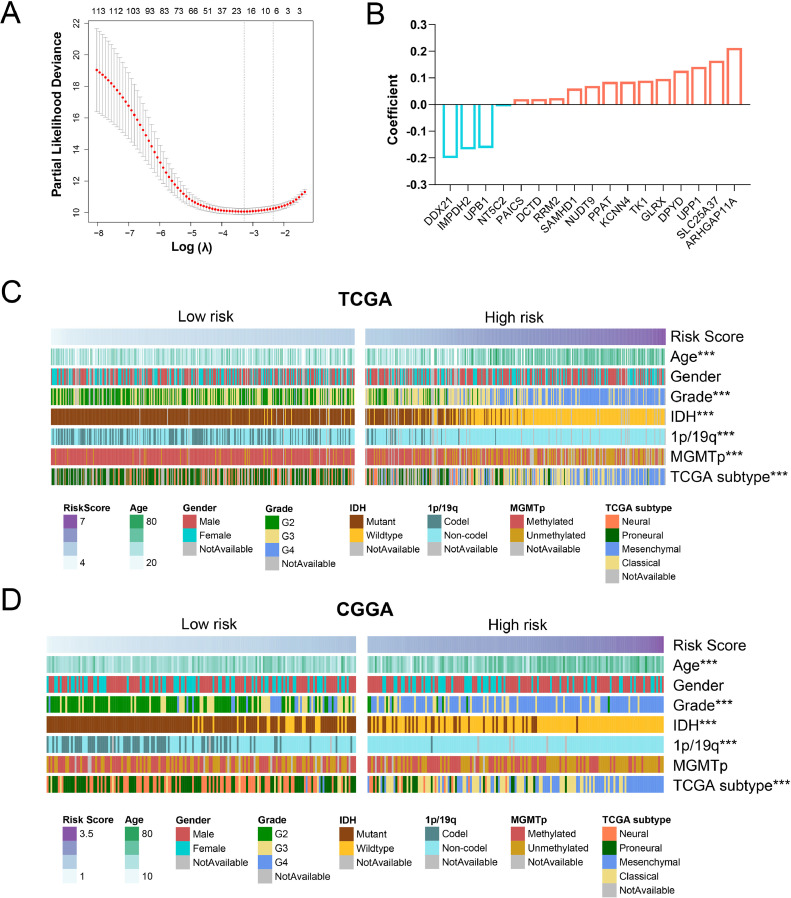
Identification of a nucleotide metabolism-related signature in gliomas. (A) Partial likelihood deviance plot of 115 nucleotide metabolism-related genes in LASSO regression analysis. (B) 17 genes selected by LASSO and their corresponding regression coefficients. (C-D) Heatmaps show the clinical and pathological features of the high-risk and low-risk groups in TCGA and CGGA datasets. ***P < 0.001. IDH, isocitrate dehydrogenase; MGMTp, methylguanine methyltransferase promoter.

To identify a nucleotide metabolism-related signature, 115 genes associated with overall survival were screened by univariate Cox regression analysis in TCGA dataset. By LASSO algorithm, the 17 most valuable predictive genes were selected as active covariates (Fig. 2A). Our nucleotide metabolism-related signature (risk score) was identified with a linear combination of expression of 17 genes weighted by their regression coefficients (Fig. 2B). Based on the median risk score as the cutoff value, we divided TCGA patients into high-risk and low-risk groups and compared clinical and pathological differences between the two groups (Fig. 2C). We found that the high-risk group was strongly linked with older age, GBM (glioblastoma, WHO grade 4), IDH-wildtype, 1p/19q non-codeletion, MGMT promoter unmethylation and classical or mesenchymal subtypes (*p* < 0.001). These findings were validated in three other three datasets (Fig. 2D and Figure S1).

### Association between nucleotide metabolism-related signature and pathological features in gliomas

**Fig. 3:**
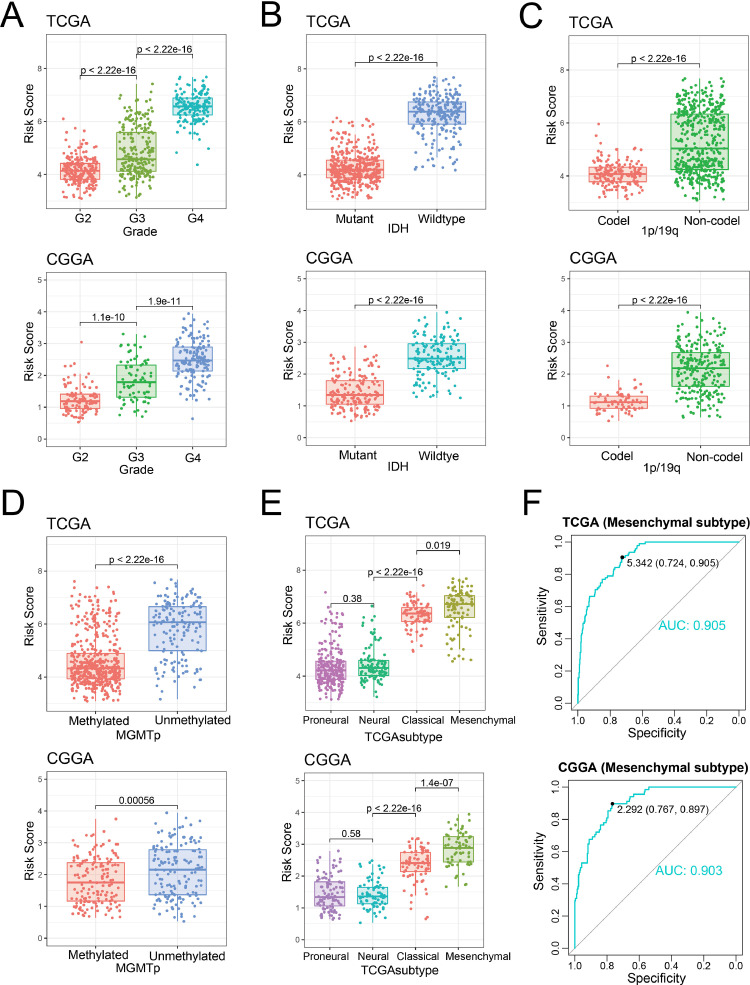
Association between the nucleotide metabolism-related signature and pathological features in gliomas. (A-E) The distribution of signature risk score in patients stratified by WHO grade, IDH mutation status, 1p/19q codeletion status, MGMT promoter methylation, and TCGA molecular subtypes. (F) Receiver operating characteristic (ROC) curves show the predictive value of risk score for mesenchymal subtype.

Since gliomas contained different pathological types, we then analyzed the differences in risk score. We found that risk score increased significantly with the increase of WHO grade from 2 to 4 (*p* < 0.05, Fig. 3A). Risk score was significantly higher in patients with IDH-wildtype, 1p/19q non-codeletion, MGMT promoter unmethylation, or TERT promoter mutation (*p* < 0.05, Fig. 3B-D, Figure S2C). Similar differences in risk score were observed in the other two validation sets (Figure S2A and B). Among TCGA molecular subtypes, mesenchymal subtype, which was associated with poor prognosis, had the highest risk score (Fig. 3E and Figure S2D). This suggested that risk score might be a biomarker for mesenchymal subtype. We further evaluated signature's ability to predict the mesenchymal subtype with Receiver Operating Characteristic (ROC) curve and the area under curve (AUC) was 0.905, 0.903, 0.822 and 0.871 in four datasets, respectively (Fig. 3F and Figure S2E).

### Association between nucleotide metabolism-related signature and genomic alterations

**Fig. 4:**
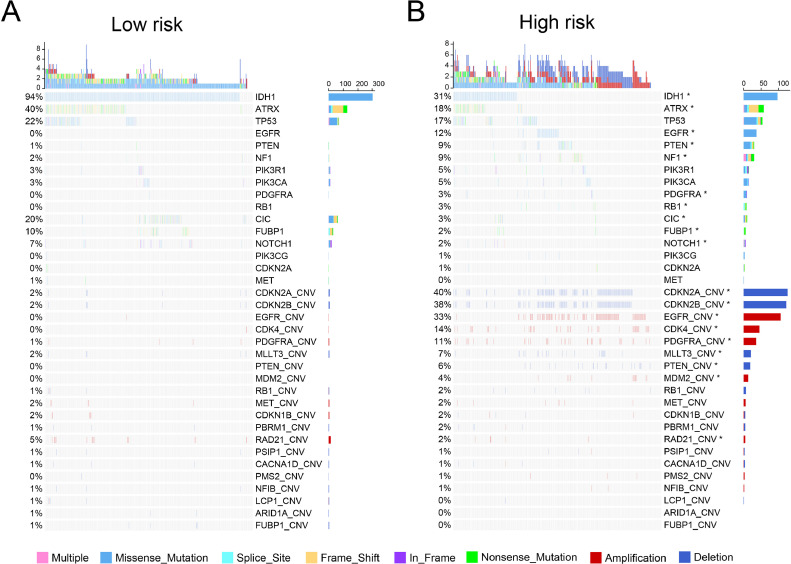
Comparison of genomic alterations between the high-risk group and low-risk group. The upper part of the oncoplots show the somatic mutations, and the lower part show copy number alterations. Chi-square test, *P < 0.05.

To reveal the molecular mechanism underlying nucleotide metabolism-related signature, we collected somatic mutation and copy-number alterations (CNA) data from TCGA dataset. In the low-risk group, we observed significant enrichment of *IDH1, ATRX, CIC, FUBP1* and *NOTCH1* mutations (*p* < 0.05, Fig. 4A). Meanwhile, high frequency of mutations in *EGFR, PTEN, NF1, PDGFRA* and *RB1* were observed in the high-risk group (*p* < 0.05, Fig. 4B). In addition to somatic mutations, we also analyzed copy number alterations. The high-risk group showed more frequently deleted regions such as CDKN2A, CDKN2B, MLLT3, PTEN, and more amplified regions such as EGFR, CDK4, PDGFRA, MDM2 (*p* < 0.05, Fig. 4). These findings suggested higher genomic alterations in the high-risk group.

### Prognostic analysis of nucleotide metabolism-related signature

**Fig. 5:**
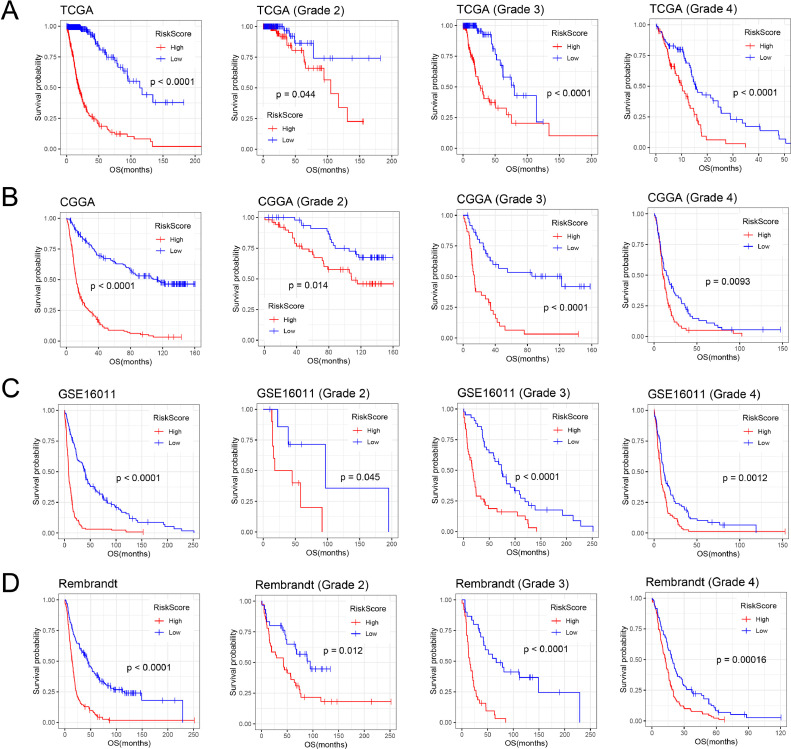
Prognostic value of the nucleotide metabolism-related signature in gliomas. Kaplan-Meier (K-M) survival curve analysis of the signature in all grade, grade 2, grade 3, and grade 4 gliomas. (A) TCGA dataset, (B) CGGA dataset, (C) GSE16011 dataset, (D) Rembrandt dataset.

To evaluate the prognostic value of nucleotide metabolism-related signature, we performed the Kaplan-Meier survival curve analysis. As shown in Fig. 5, patients in the high-risk group had significantly shorter overall survival than those in the low-risk group of four datasets (*p* < 0.001 by log-rank test). Due to the heterogeneity of different grades of gliomas, we analyzed survival curves in grade 2, grade 3, and grade 4 gliomas, and observed the same prognostic difference (*p* < 0.05 by log-rank test). In addition, univariate and multivariate Cox regression analysis showed that the signature risk score (HR, 2.589; 95 % CI, 1.649–4.063; *p* = 3.54E-05) was an independent prognostic indicator of glioma in TCGA dataset (Table 1). Similar results were shown in the other three validation sets (*p* < 0.05).

**Table 1:** Variables related to OS in gliomas: univariate and multivariate analysis.

TCGA	Univariate Cox regression			Multivariate Cox regression		
	HR	95 %CI	*p* value	HR	95 %CI	*p* value
Age, Increasing years	1.075	1.063–1.088	<2e-16*	1.058	1.034–1.082	1.13e-06*
Gender, (male vs. female)	1.001	0.743–1.347	0.997			
Grade, (GBM vs. LGG)	9.576	6.835–13.420	<2e-16*	0.720	0.338–1.531	0.393
IDH, wild vs. mutant type)	11.070	7.772–15.770	<2e-16*	3.103	1.172–8.218	0.023*
1p/19q, (non-codel vs. codel)	4.541	2.671–7.719	2.28e-08*	0.552	0.183–1.665	0.291
MGMT promoter, (unmethylated vs. methylated)	3.207	2.312–4.447	2.88e-12*	1.089	0.571–2.075	0.796
TERT promoter, (mutant vs. wild type)	2.075	1.312–3.281	0.002*	0.458	0.186–1.130	0.090
RiskScore, Increasing scores	3.597	3.040–4.256	<2e-16*	2.589	1.649–4.063	3.54e-05*

CGGA	Univariate Cox regression			Multivariate Cox regression		
	HR	95 %CI	*p* value	HR	95 %CI	*p* value
Age, Increasing years	1.035	1.023–1.048	2.71e-08*	1.011	0.998–1.024	0.087
Gender, (male vs. female)	0.998	0.759–1.312	0.988			
Grade, (GBM vs. LGG)	4.919	3.670–6.593	<2e-16*	2.098	1.479–2.978	3.33e-05*
IDH, (wild vs. mutant type)	2.866	2.171–3.782	1.05e-13*	0.639	0.431–0.948	0.026*
1p/19q, (non-codel vs. codel)	5.877	3.602–9.588	1.33e-12*	2.904	1.700–4.959	9.48e-05*
MGMT promoter, (unmethylated vs. methylated)	1.195	0.911–1.566	0.199			
RiskScore, Increasing scores	3.210	2.670–3.860	<2e-16*	2.272	1.712–3.014	1.29e-08*

GSE16011	Univariate Cox regression			Multivariate Cox regression		
	HR	95 %CI	*p* value	HR	95 %CI	*p* value
Age, Increasing years	1.041	1.030–1.051	4.19e-14*	1.041	1.024–1.060	4.77e-06*
Gender, (male vs. female)	1.066	0.811–1.401	0.647			
Grade, (GBM vs. LGG)	3.131	2.353–4.166	4.92e-15*	1.141	0.613–2.125	0.678
IDH, (wild vs. mutant type)	1.930	1.423–2.618	2.34e-05*	1.300	0.769–2.198	0.328
1p/19q, (non-codel vs. codel)	2.445	1.645–3.633	9.68e-06*	1.330	0.801–2.210	0.270
RiskScore, Increasing scores	2.838	2.286–3.524	<2e-16*	2.244	1.291–3.900	0.004*

Rembrandt	Univariate Cox regression			Multivariate Cox regression		
	HR	95 %CI	*p* value	HR	95 %CI	*p* value
Gender, (male vs. female)	1.105	0.828–1.475	0.496			
Grade, (GBM vs. LGG)	2.671	2.060–3.464	1.24e-13*	1.669	0.978–2.848	0.060
1p/19q, (non-codel vs. codel)	2.348	1.287–4.285	0.005*	1.564	0.695–3.521	0.280
RiskScore, Increasing scores	4.052	3.053–5.377	<2e-16*	2.509	1.313–4.795	0.005*

HR, hazard ratio; CI, confidence interval.

⁎ Significant.

### Construction of an individualized prediction model based on the signature

**Fig. 6:**
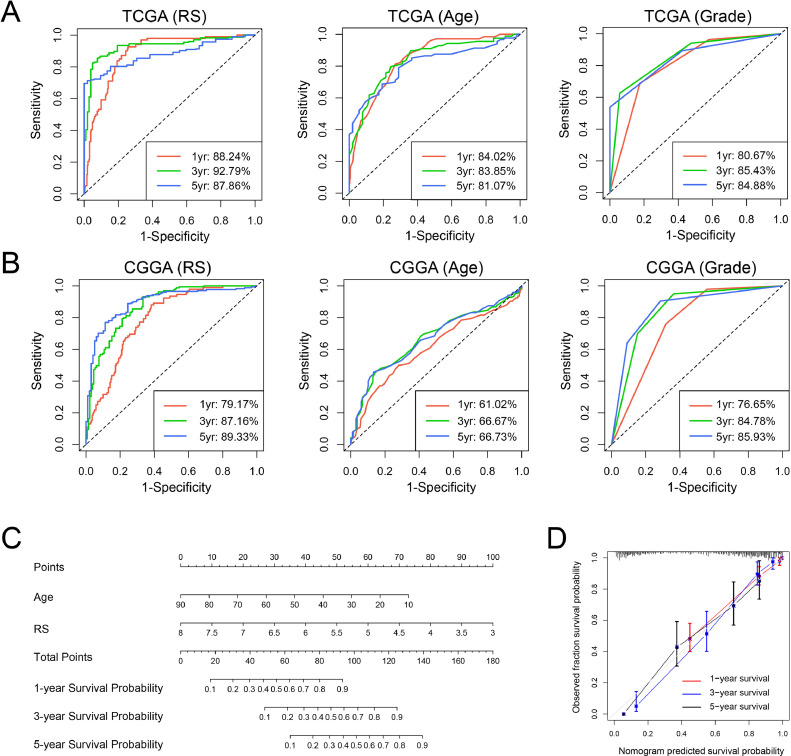
A nomogram model for predicting the survival of glioma patients. (A-B) The timeROC curves to predict 1-year, 3-year, and 5-year overall survival of glioma patients based on risk score (RS), age, and grade in TCGA and CGGA dataset. (C) A nomogram model was constructed by integrating the signature RS and age in TCGA dataset. (D) Calibration curve of the nomogram model for predicting 1-year (red line), 3-year (blue line) and 5-year (black line) overall survival.

To assess the predictive value of the signature for overall survival, we performed the timeROC analysis. The 1-year, 3-year and 5-year AUC of our signature risk score were 88.24 %, 92.79 %, and 87.86 %, superior to age (84.02 %, 83.85 %, 81.07 %) and grade (80.67 %, 85.43 %, 84.88 %) in TCGA dataset (Fig. 6A). We found the similar results in the other three validation sets (Fig. 6B and Figure S3). Next, we constructed a nomogram model to evaluate individual prognosis by integrating independent prognostic indicators (age and risk score) of TCGA dataset (Fig. 6C). The concordance index (C-index) of our nomogram was 0.867 and the calibration curve showed a satisfactory agreement between actual observations and model predictions (Fig. 6D).

### Functional annotation of nucleotide metabolism-related signature

**Fig. 7:**
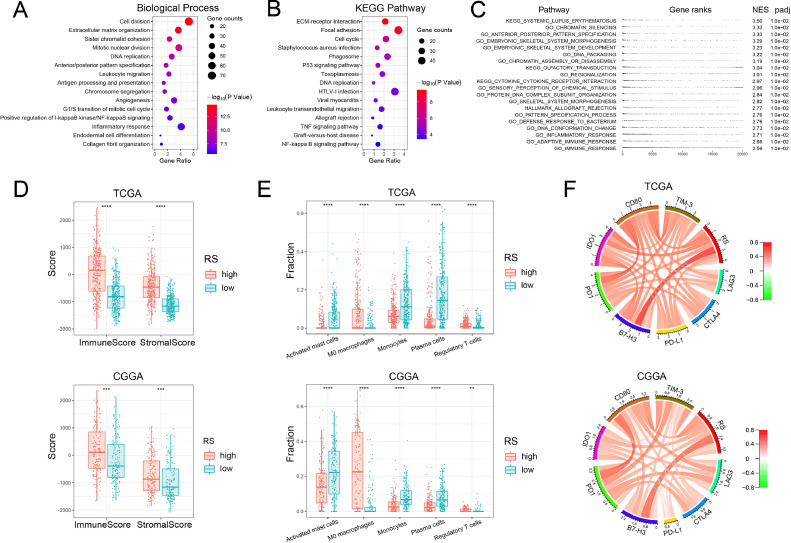
Functional and immune analysis of the nucleotide metabolism-related signature in gliomas. (A) GO analysis for genes positively correlated with signature. (B) KEGG pathway analysis for genes positively correlated with signature. (C) GSEA analysis revealed biological functions and pathways that were enriched in the high-risk group. (D) ESTIMATE analysis shows differences in immune scores and stromal scores between the high-risk and low-risk groups. (E) CIBERSORT analysis shows differences in the proportion of different immune cell types between the high-risk and low-risk groups. (F) Pearson correlation analysis of immune checkpoint gene expression and risk score. **P < 0.01, ***P < 0.001, ****P < 0.0001.

In order to explore the potential function of nucleotide metabolism-related signature, we first performed Pearson correlation analysis to determine genes that were positively correlated with signature (*R* > 0.6, *p* < 0.05). GO analysis showed that these genes were mainly involved in biological processes including cell division, mitotic nuclear division, DNA replication, chromosome segregation and immune response (such as leukocyte migration, antigen processing and presentation, inflammatory response) (Fig. 7A). And KEGG pathway analysis showed that these genes were enriched in pathways including cell cycle, p53 signaling pathway, DNA replication and immune response (such as leukocyte transendothelial migration, TNF signaling pathway, NF-kappa B signaling pathway) (Fig. 7B). Meanwhile, GSEA analysis showed that similar functions were enriched in the high-risk group (Fig. 7C). These results suggested that nucleotide metabolism was not only related to cell division and cell cycle, but also affected immune response in gliomas.

### Association between nucleotide metabolism-related signature and glioma immunity

To further understand the relationship between nucleotide metabolism and glioma immunity, we first calculated the immune score and stroma score for each glioma sample by ESTIMATE algorithm. We found that the immune score and stromal score of the high-risk group were significantly higher than those of the low-risk group (*p* < 0.05, Fig. 7D). Then, we performed CIBERSORT algorithm to quantitatively analyze the proportion of each immune cell subtype. We observed that resting or suppressive immune cells, including M0 macrophages and regulatory T cells, were significantly enriched in the high-risk group (*p* < 0.05, Fig. 7E). While activated immune cells, including activated mast cells, monocytes, and plasma cells, were significantly enriched in the low-risk group (*p* < 0.05). In addition, we selected representative immune checkpoint genes and signature risk score for Pearson correlation analysis. Chord diagrams showed positive correlations between risk score and the expression of immune checkpoint genes, suggesting that gliomas in the high-risk group might be immunosuppressive (Fig. 7F).

To better reveal the differences in the microenvironments of the two groups, single cell RNA sequencing (scRNA-seq) was performed on two IDH-wildtype glioblastomas. One sample had a high risk score (RS-high) and the other a low risk score (RS-low). Based on canonical markers for each cell type (Figure S4C), we identified four cell types, including tumor cells, macrophages, lymphocytes, and oligodendrocytes (Figure S4A and B). In RS-high samples, we confirmed a significantly higher proportion of tumor-infiltrating immune cells (including macrophages and lymphocytes, Figure S4D). And we preliminarily analyzed the functional phenotype of macrophages and found that the abundant macrophages in RS-high samples were mainly M2-like (anti-inflammatory, Figure S4E), while the macrophages in low-risk samples were mainly M1-like (pro-inflammatory, Figure S4F). This further confirmed the immunosuppressive status of tumors in the high-risk group.

## Discussion

Metabolic changes in tumors have been observed for nearly a century, but in recent years the study of metabolism in cancer has gained renewed interest. Metabolic reprogramming is ubiquitous in cancer, giving tumor cells the ability to survive and proliferate under stress conditions [6]. Common metabolic reprogramming in cancer includes glucose metabolism, lipid metabolism, amino acid metabolism, nucleotide metabolism and other bioenergy metabolism pathways [26]. In proliferating tumor cells, increased nucleotide synthesis is required for DNA replication and RNA production to support protein synthesis during the cell cycle. Nucleotide metabolism and factors affecting this process have been confirmed to be involved in the development and progression of cancer [4]. Minakshi et al. found that the lncRNA lincNMR affected tumor cell proliferation by regulating nucleotide metabolism [27]. Madhusudhan et al. reported that mutated p53 drives and maintains cancer development by controlling nucleotide synthesis [28]. In glioblastoma stem cells, Wang et al. revealed that high levels of pyrimidine metabolism were necessary to maintain the stem-like phenotype that promoted self-renewal and tumorigenesis [29]. Norihiro et al. found that driving the synthesis of pyrimidine nucleotide enhanced the liver metastasis of colorectal cancer [30]. In this study, we detected the nucleotide metabolic status of gliomas, and for the first time developed a nucleotide metabolism-related signature through comprehensive analysis of transcriptome data. We divided gliomas into high-risk or low-risk groups based on median signature, and found significant enrichment of older age, WHO grade 4, IDH-wildtype, 1p/19p intact, MGMT promoter unmethylation and mesenchymal subtype in the high-risk group. And ROC curve showed that our signature was a potential biomarker for mesenchymal subtype. Using the survival curve and univariate and multivariate Cox regression analysis, we found that signature was an independent prognostic factor of gliomas. This suggested that nucleotide metabolism might influence the prognosis of glioma patients. Combining signature and age, we further constructed a nomogram model to predict individual survival.

In addition to clinical and pathological differences, we performed an integrated analysis of genomic alterations. We found that mutations of *EGFR, PTEN, NF1, PDGFRA* and *RB1* were more common in the high-risk group, while mutations of *IDH1, ATRX, CIC, FUBP1* and *NOTCH1* were more common in the low-risk group. In addition to gene mutation differences, we also found that copy number variation was more frequent in the high-risk group. These results revealed the relationship between genomic alterations and nucleotide metabolism in gliomas, suggesting that genomic alterations might be the root cause of nucleotide metabolic reprogramming in gliomas.

Functional analysis showed that signature was not only associated with cell division and cell cycle but also with immune responses, suggesting an interaction between nucleotide metabolism and immune environment. Recent studies have shown that abnormal nucleotide metabolism not only supports tumor cell growth but also regulates immune responses in tumor microenvironment. Purine nucleotides affect the cytotoxicity of immune cells by regulating the expression of immune ligand MICA [31]. Adenosine enrichment in tumors decreased the infiltration of protective immune cells, including T cells and NK cells, while enhancing the role of immunosuppressive subsets, including regulatory T cells and MDSCs [32]. Inhibition of purine nucleotide synthesis in tumor can increase the expression of immunoproteasome and enhance the response of CD8 T cells to anti-PD-1 immunotherapy [33]. Regulation of nucleotide metabolism may potentially improve the response to tumor immunotherapy and immunosuppressive state in tumor, consequently inducing tumor cell death [34]. To further understand the relationship between nucleotide metabolism and glioma immunity, we first performed ESTIMATE algorithm and found that the immune score and stromal score of the high-risk group were significantly higher than those of the low-risk group. And CIBERSORT analysis revealed that immunosuppressive subsets, including M0 macrophages and regulatory T cells, were enriched in the high-risk group. Pearson correlation analysis showed that the expression of immune checkpoint genes was positively correlated with risk score. These results suggested that nucleotide metabolites might regulate the infiltration of different types of immune cells and were associated with immunosuppressive status in gliomas.

## Conclusions

In summary, we comprehensively analyzed the expression of nucleotide metabolism-related genes in gliomas and developed a nucleotide metabolism-related signature for prognostic stratification of patients with gliomas. Our findings help extend our understanding of nucleotide metabolism and provide an important reference for nucleotide metabolism-targeted therapy in gliomas.

## CRediT authorship contribution statement

**Ai-Qin Chen:** Writing – review & editing, Writing – original draft, Supervision, Project administration, Formal analysis, Data curation, Conceptualization. **Qi-Xuan Jiang:** Visualization, Validation, Software, Resources, Methodology, Formal analysis, Data curation. **Yong-Jian Zhu:** Writing – review & editing, Validation, Software, Resources, Methodology, Investigation, Funding acquisition. **Qiang-Wei Wang:** Writing – review & editing, Writing – original draft, Supervision, Software, Resources, Investigation, Formal analysis, Data curation.

## Declaration of competing interest

The authors declare that they have no known competing financial interests or personal relationships that could have appeared to influence the work reported in this paper.

## Appendix Supplementary materials

Image, application 1
