# Supplementary material for: Transcriptomic profiling identifies a nucleotide metabolism-related signature with prognostic power in gliomas
Source: Transl Oncol. 2024 Aug 8;49:102068. doi: 10.1016/j.tranon.2024.102068 (PMC11362638; doi:10.1016/j.tranon.2024.102068)

**Figure S1. Heatmap and clinicopathological features of low-risk and high-risk group in GSE16011 and Rembrandt dataset.** ***P < 0.001.


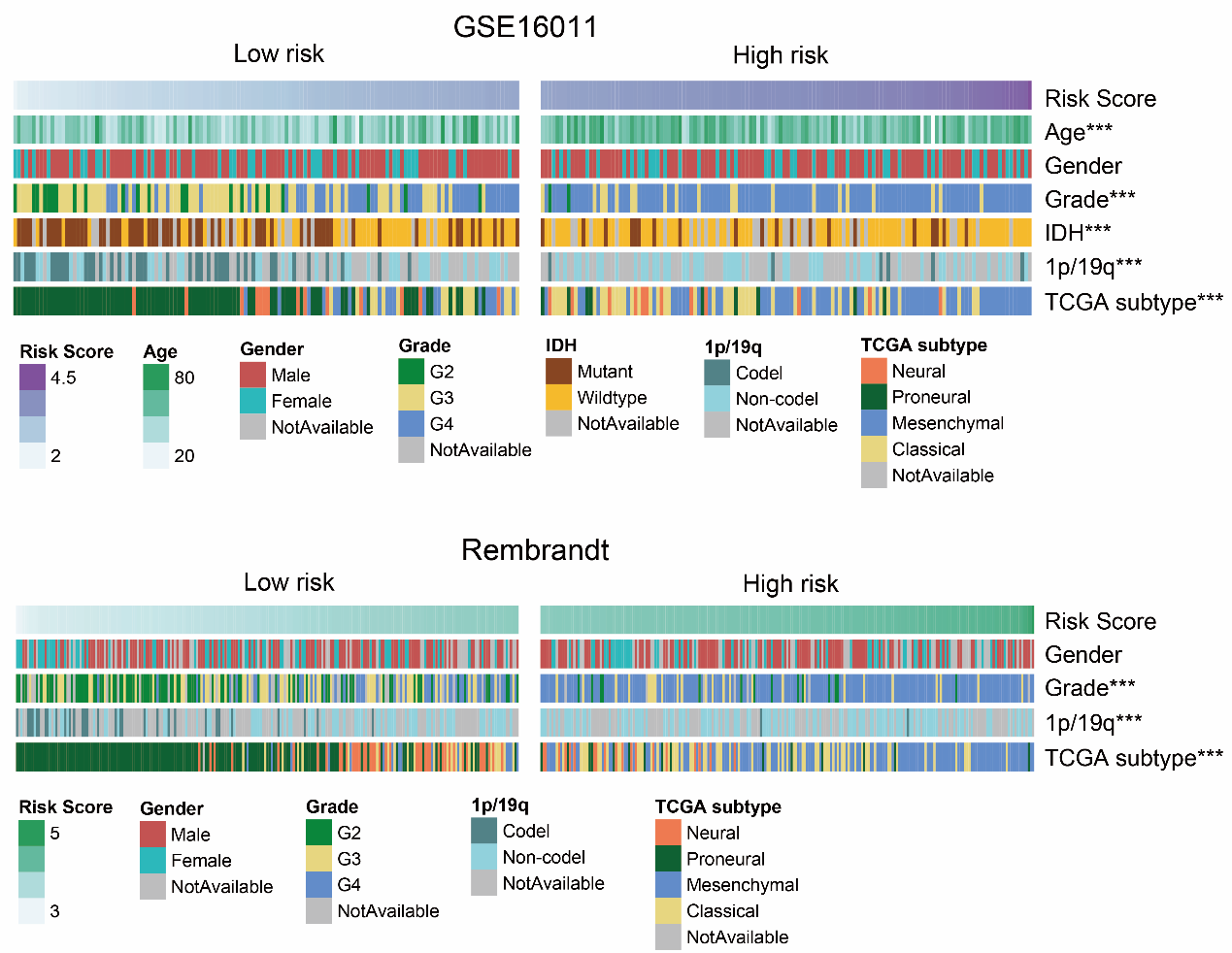


**Figure S2**. **Associations between the nucleotide metabolism-related signature and pathological features in GSE16011 and Rembrandt dataset.** (**A, B, D**) Patients were grouped by WHO grade, IDH mutation status, 1p/19q codeletion status, TCGA molecular subtype. (**E**) The ROC curve evaluated the predictive value of risk score for mesenchymal subtype. (**C**) The distribution of signature risk score in patients stratified by TERT promoter mutation status in TCGA dataset.


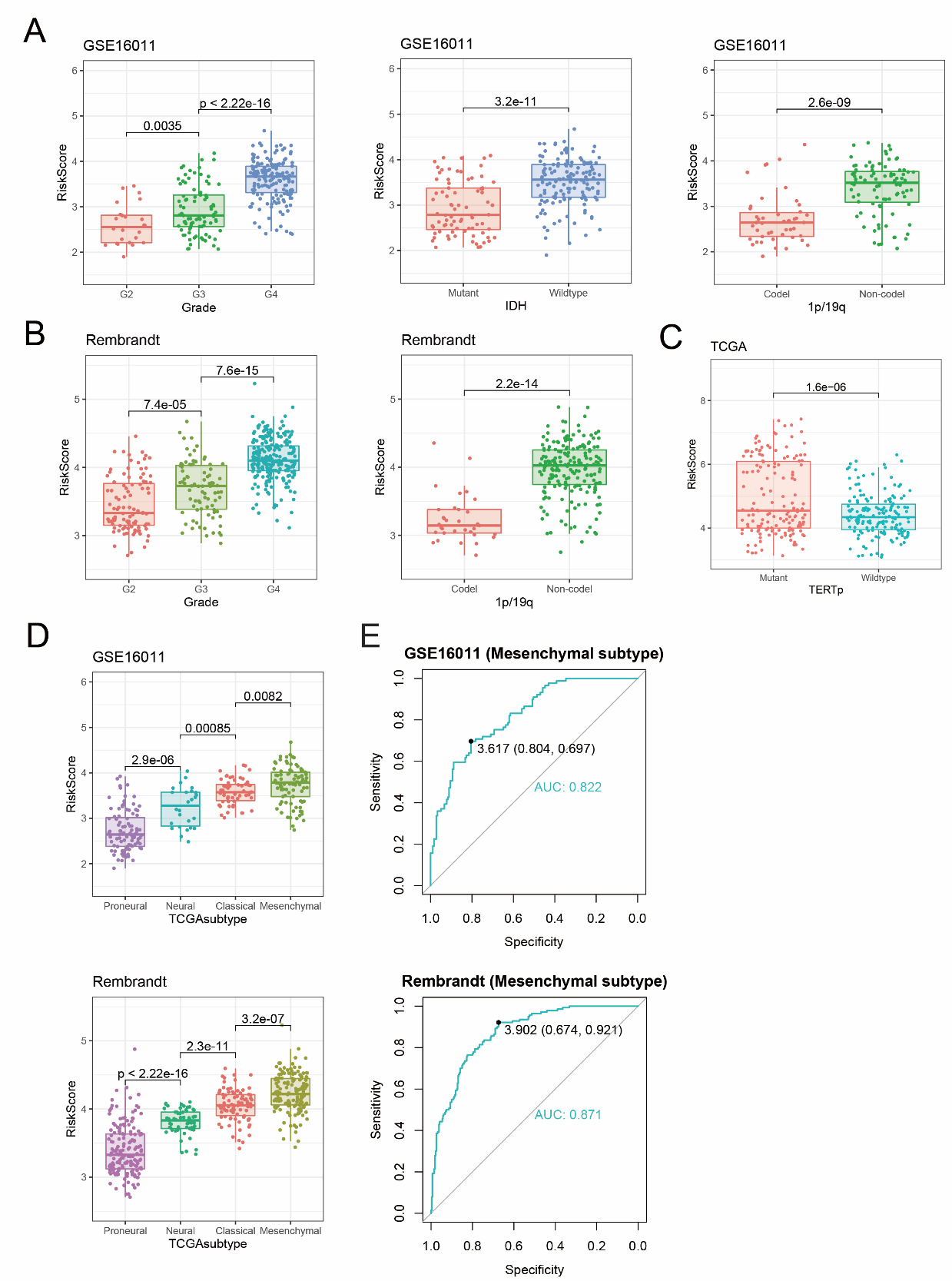


**Figure S3. Predictive value of signature, age and grade for overall survival in GSE16011 (A) and Rembrandt dataset (B).** 1-year, 3-year and 5-year ROC curves indicated the sensitivity and specificity of risk score, age and grade.


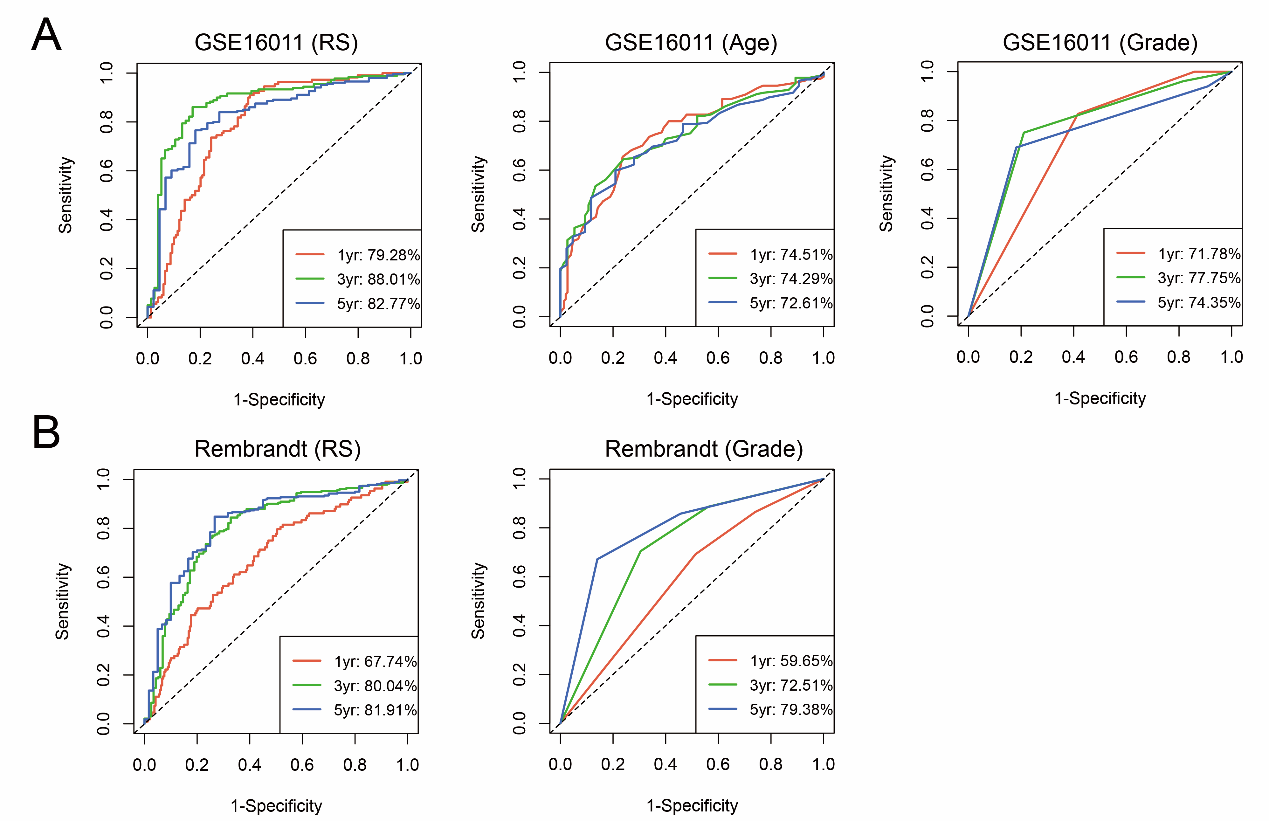


**Figure S4. Single-cell transcriptome analysis of primary IDH-wildtype GBM. (A)** Cells were grouped into four major cell types from two glioma patient samples. One sample had a high risk score (RS-high) and the other a low risk score (RS-low). (B) Distribution of cell types in two samples. (C) Dot plot showing the gene-expression patterns of classical cell-type marker genes. (D) Bar plot showing the cell-type fraction in two samples. Expression of M2-like marker genes (E), and M1-like marker genes (F) in macrophages from two samples.


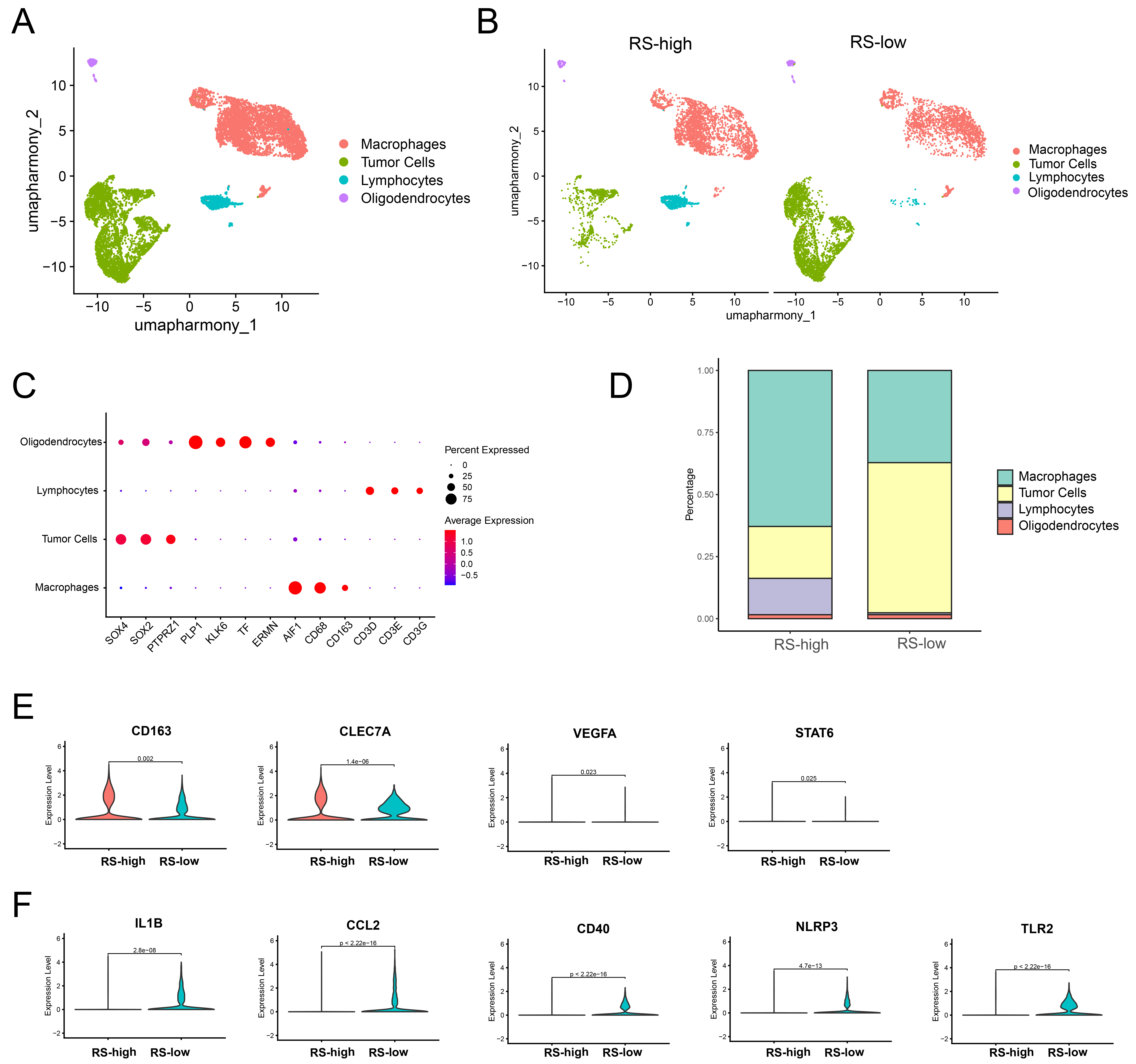

Supplement: Supplementary file 1 [file mmc1.docx]
